# Fruquintinib in Combination With PD-1 Inhibitors in Patients With Refractory Non-MSI-H/pMMR Metastatic Colorectal Cancer: A Real-World Study in China

**DOI:** 10.3389/fonc.2022.851756

**Published:** 2022-07-07

**Authors:** Miaomiao Gou, Niansong Qian, Yong Zhang, Huan Yan, Haiyan Si, Zhikuan Wang, Guanghai Dai

**Affiliations:** ^1^ Medical Oncology Department, The Fifth Medical Center, Chinese People’s Liberation Army General Hospital, Beijing, China; ^2^ Sanya Medical Center, Chinese People’s Liberation Army General Hospital, Sanya, China; ^3^ Medical Oncology Department, The Second Medical Center, Chinese People’s Liberation Army General Hospital, Beijing, China

**Keywords:** fruquintinib, PD-1 inhibitors, mCRC, real-world study, non-MSI-H/pMMR

## Abstract

**Background:**

Fruquintinib, a vascular endothelial growth factor receptor inhibitor, is a new anticancer drug independently developed in China to treat refractory metastatic colorectal cancer (mCRC). In Japan, regorafenib combined with nivolumab has been demonstrated to be promising in patients with refractory mCRC. Here, in a real-world study, we were aimed to evaluate the efficacy of fruquintinib with various programmed death-1 (PD-1) inhibitors after standard treatment in Chinese non-microsatellite instability-high (MSI-H)/mismatch repair proficient mCRC patients.

**Methods:**

A total of 45 patients with refractory mCRC were involved in the study. They received fruquintinib (3 or 5 mg, orally administered once a day for 3 weeks followed by 1 week off in 4-week cycles) and a PD-1 inhibitor(200 mg pembrolizumab, 3 mg/kg nivolumab, 200 mg sintilimab or camrelizumab, intravenously administered on D1 once every 3 weeks). Progression-free survival (PFS), overall survival (OS), disease control rate (DCR), and objective response rate (ORR) were reviewed and evaluated.

**Results:**

Among the 45 patients, the median age was 54 years (29-85). The ORR was 11.1% (5/45), DCR 62.2% (28/45), median PFS equal 3.8 months, and median OS was 14.9 months. The response duration was 3.4 months. PFS between left and right primary tumors and PFS with or without lung metastases were both not significantly different (*p* > 0.05), which was inconsistent with the result of REGONIVO study. The multivariate analysis indicated no association of OS benefit in the specified subgroups. No adverse-effect-related deaths were reported.

**Conclusions:**

Fruquintinib, in combination with anti-PD-1, was observed to have clinical activity in a small population of patients with heavily pretreated mCRC in our center. Further studies are needed to verify this outcome in a large population.

## Introduction

Colorectal cancer (CRC) is the fourth most common cancer and the fifth leading cause of cancer-related deaths in China (1). Despite of the improvements of treatments for advanced or metastatic CRC, their survival following one or two previous standard lines of therapy remains dismal. Currently, the United States Food and Drug Administration has approved TAS-102 and regorafenib as third-line therapies for metastatic CRC (mCRC) patients (2, 3). As reported by the CONCUR (4) and TERRA studies (5), the average progression-free survival (PFS) was only three months in Asians. Consequently, attempts have been made to improve the survival benefit. In 2019, the REGONIVO study (6) demonstrated the encouraging activity and manageable safety of nivolumab plus regorafenib in patients with late-stage microsatellite stability (MSS) or mismatch repair proficient (pMMR) gastric cancer and CRC. Regorafenib (7) is a novel oral multi-kinase inhibitor that blocks the activity of several protein kinases, including those involved in the regulation of tumor angiogenesis (vascular endothelial growth factor receptor [VEGFR]1, VEGFR2, VEGFR3, and TIE2) and oncogenesis (KIT, RET, RAF1, BRAF, and BRAFV600E). On the other hand, nivolumab is a human immunoglobulin G(4)-blocking antibody that inhibits the T-cell programmed death-1 (PD-1) checkpoint protein. Nivolumab, whether alone or combined with other agents, has demonstrated its encouraging role in tumor control in several cancers (8–11). The combination of a PD-1 inhibitor with angiogenesis agents might have a potential in tumor control, as suggested by the REGONIVO study. Despite of that, the immunotherapy-based options for mCRC harboring MSS phenotype remain dismal (12, 13), with PFS around 3-4months in China and even worldwide (14–16).

Fruquintinib, an oral multi-kinase inhibitor, is a potent, highly selective small-molecule inhibitor of VEGFR1, VEGFR2, and VEGFR3. Based on the results of FRESCO study and according to the guidelines of Chinese Society of Clinical Oncology in 2019, fruquintinib has been recommended as a treatment in Chinese mCRC patients who experienced tumor progression following two or more prior chemotherapy regimens (17, 18). Now, at least six types of PD-1 inhibitors are available in clinical practice. However, no existing data on the effectiveness of fruquintinib in conjunction with anti-PD-1 inhibitors for MSS/pMMR mCRC has been found in a real-world situation. Here, we sought to further explore the efficacy of fruquintinib plus PD-1 inhibitors and the potential correlation of clinical benefits in patients with metastatic MSS/pMMR CRC that had progressed following second-line or subsequent treatment retrospectively in our clinic center.

## Patients and Methods

Patients who progressed to conventional treatment at the cancer department of Chinese People’s Liberation Army General Hospital from January 2019 to January 2022 were included. The inclusion criteria were: (i) Patients with histological or cytological confirmation of adenocarcinoma of the colon or rectum; (ii) Patients who have failed from first and second standard therapies such as FOLFOX (fluoropyrimidine and oxaliplatin) or FORFIRI (irinotecan and fluoropyrimidine) with or without bevacizumab or cetuximab; (iii) Patients who have at least one non-resectable measurable lesion to evaluate treatment response; and (iv) Patients who were administered at least two cycles of treatment. The exclusion criteria included: (i) Patients with less than one cycle of treatment and (ii) Patients with little information on tumor response. This retrospective study was approved by the independent ethics committee of Chinese People’s Liberation Army General Hospital (NO: S2019-201-01).

Fruquintinib was orally administered once a day in a 28-day (D) cycle (21D on/7D off). The PD-1 inhibitor (200 mg pembrolizumab, 3 mg/kg nivolumab, 200 mg sintilimab, or camrelizumab) was intravenously administered on D1 once every 3 weeks. The starting dose of fruquintinib was 5 mg, and could be reduced to 3 or 4 mg later in the therapy cycle if not well tolerated.

### Assessments

Patients were followed up until the cutoff date of January 2022. Tumor evaluation was based on RECIST (version 1.1). Moreover, the response evaluation included complete response (CR), partial response (PR), stable disease (SD), and progression disease (PD). In addition, the objective response rate (ORR) and disease control rate (DCR) were also evaluated. The ORR was calculated as the sum of CR and PR, while the DCR was the sum of CR, PR and SD.

The primary endpoint was PFS, defined as the time from treatment to the RECIST-defined disease progression. The second endpoint was the duration of response (DOR), the time from the first response to disease progression or death from any cause, whichever occurred first. Finally, the overall survival (OS) was the time from third-line treatment to death for any reason.

Exploratory univariate analyses were performed using the log-rank test, which compared different clinical variables such as tumor location (left versus right side), KRAS status (wild or mutant), and liver and lung metastases.

### Statistical Analysis

SPSS software 18.0 was used for statistical analysis. Objective response and disease control were presented as proportions, and the stratified k-square test was carried out for comparisons between groups. In addition, Kaplan-Meier curves were used to estimate the median of OS, PFS, and DOR. Differences between clinic features in PFS and OS were assessed using the log-rank test. The statistical significance level was set to 0.05, and confidence intervals (CIs) were 95%.

## Results

A total of consecutive 45 patients who visited our hospital’s oncology department between January 2019 and January 2022 were enrolled in this retrospective study. Clinical characteristics of the patients were listed in **Table 1**. The average age was 54 years old (range 29-85), and 73.3% had ECOG performance status (PS) scores of 0/1. Liver and lung metastases were observed in 80% and 42.2% of the patients, respectively. Besides, 68.9% of the primary tumors were located in the left and 31.1% were located in the right colon. In addition, 88.9% of patients had received bevacizumab before treatment. Twenty-four patients had KRAS mutations, and all patients achieved MSS. The patients represented all of the patients who received the regimen.

**Table 1:** Baseline Characteristics of patients (n=45).

Characteristics	No. patients	%
Total	45	
Gender		
Male	30	66.7%
Female	15	33.3%
Age median =54		
<54	21	46.7%
>=54	24	53.3%
ECOG PS		
0-1	33	73.3%
>=2	12	26.7%
Tumor location		
left	31	68.9%
right	14	31.1%
Histological differentiation		
Poorly	2	4.4%
Moderately	41	91.1%
Well	2	4.4%
Kras status		
Wild	21	46.7%
Mutant	24	53.3%
Number of metastatic organs		
<=2	25	55.6%
>2	20	44.4%
Liver metastasis		
Yes	36	80.0%
No	9	20.0%
Lung metastasis		
Yes	19	42.2%
No	26	57.8%
Prior surgery		
Yes	33	73.3%
No	12	26.7%
Prior Bevacizumab		
Yes	40	88.9%
No	5	11.1%

The median number of cycles was 5.0 (range 2–24). Due to the tolerance, 4 patients had to deescalate fruquintinib from 5 mg to 3 mg. Six patients started fruquintinib at 3 mg; one discontinued the treatment because of an adverse event. Finally, 60.0% (27/45), 15.6%(7/45), 6.7% (3/45) and 17.8%(8/45) of patients received fruquintinib in combination with sintilimab, pembrolizumab, nivolumab, and camrelizumab, respectively. and also comment

**Figure 1:**
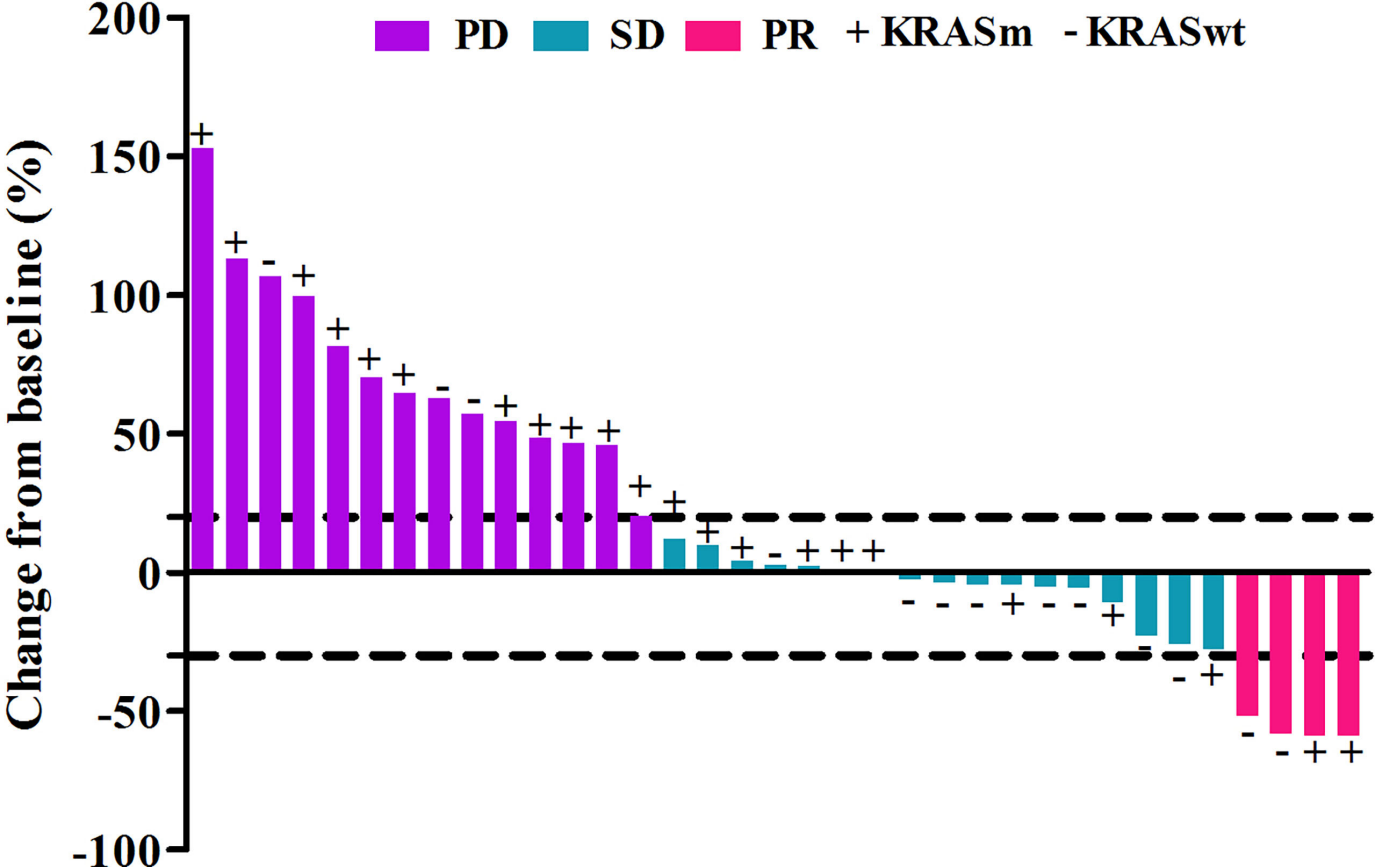
Tumor response in patients treated with fruquintinib plus pd-1 inhibitor with different kras status.

**Figure 2:**
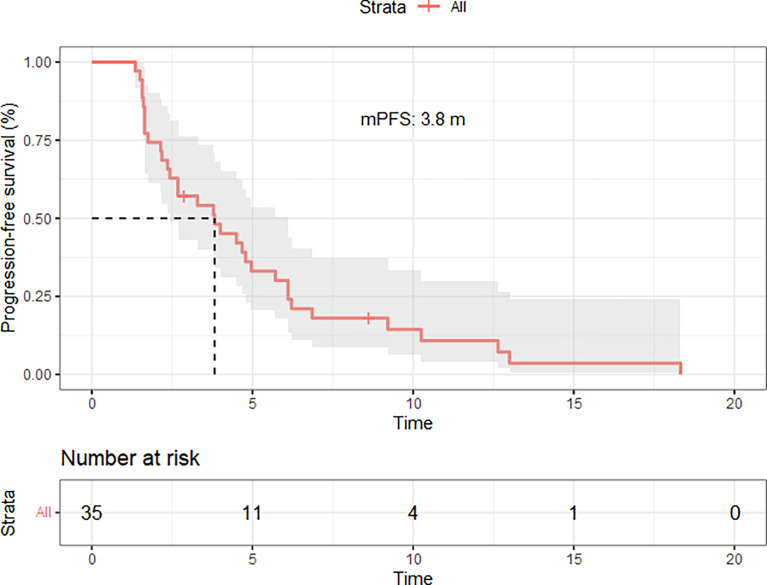
Progression free disease of patients treated with fruquintinib plus pd-1 inhibitor.

**Figure 3:**
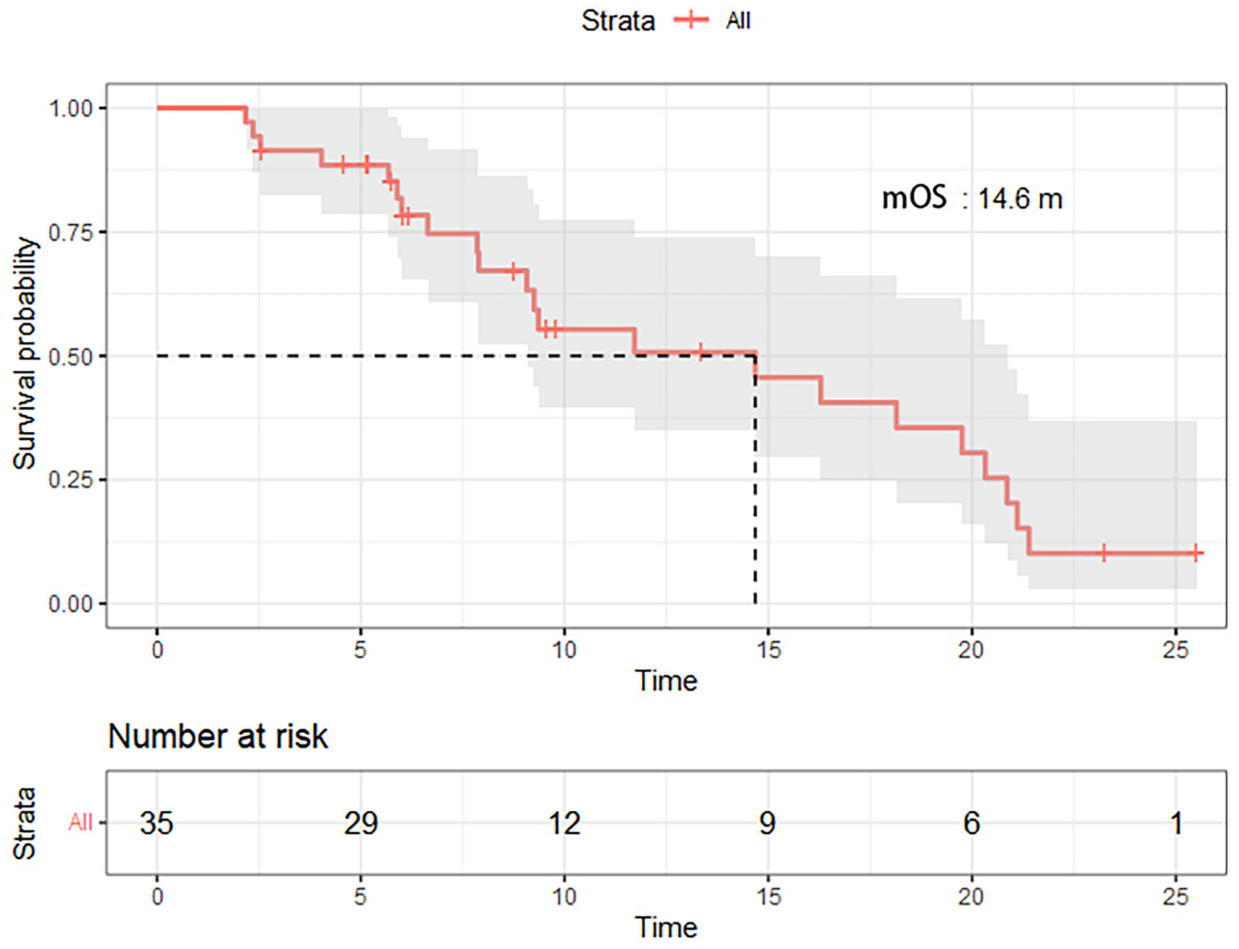
Overall survival of patients treated with fruquintinib plus pd-1 inhibitor.

**Figure 4:**
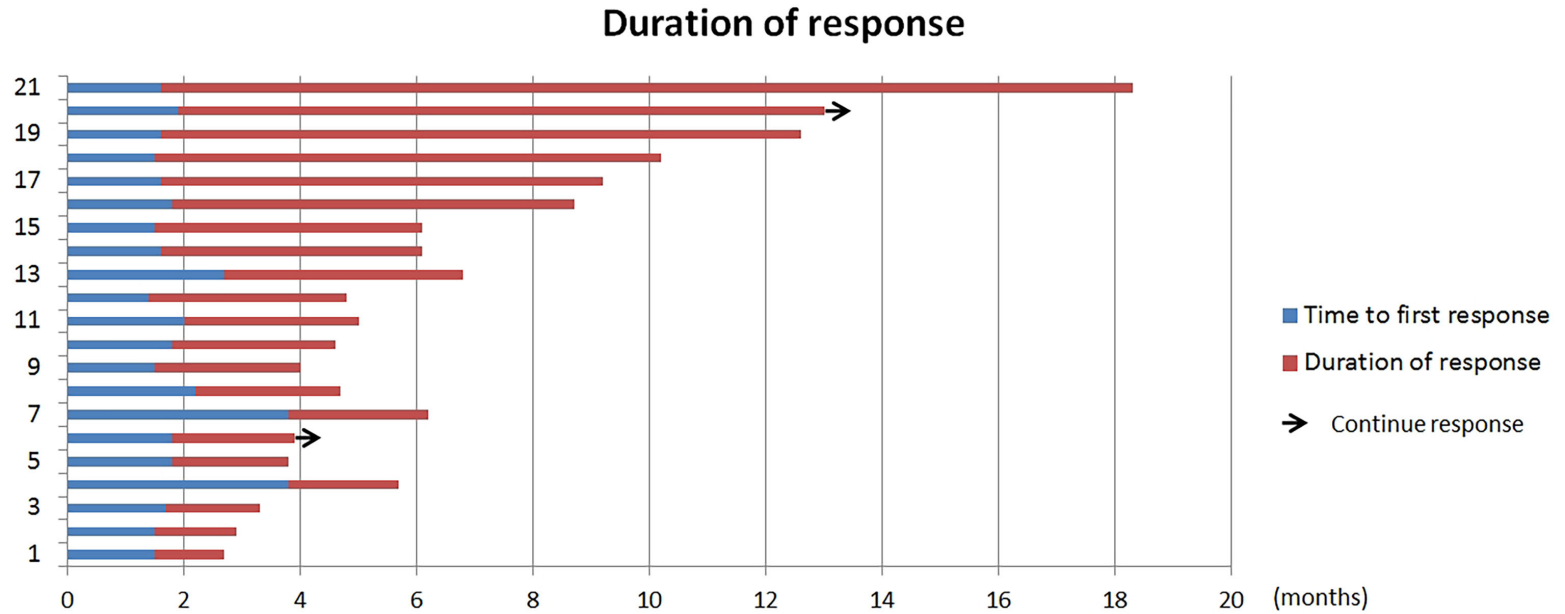
Duration of time of patients treated with fruquintinib plus pd-1 inhibitor.

**Figure 5:**
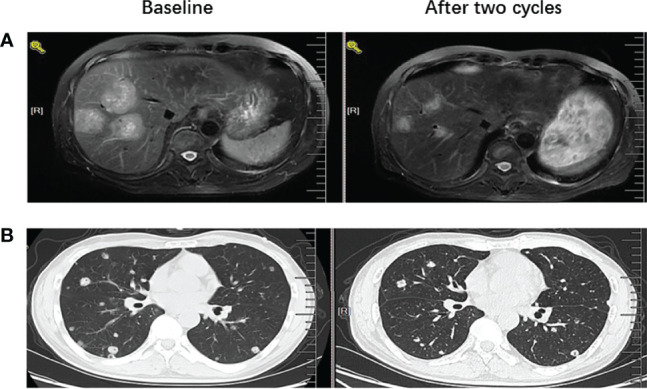
MRI or CT images before and after treatment to show PR and SD results **(A)** left image showed baseline MRI images for one patient with liver metastases before the treatment with fruquintinib plus pd-1 inhibitor and right image showed the tumor reduction more than 50% after two cycles of treatment. **(B)** left image showed baseline CT images for one patient with lung metastases before the treatment with fruquintinib plus pd-1 inhibitor and right image showed the tumor remain stable after two cycles of treatment.

Out of the 45 patients, no patient achieved a CR. Five patients achieved PR, 23 patients had SD, and 17 achieved PD following RECIST version 1.1 by centralized confirmation of imaging response data as to the consistency of the data which were collected. DCR was 62.2%, and ORR was 11.1% (**Table 2**; **Figure 1**). The median PFS was 3.8 months (95% CI: 2.8–4.8 months), and the OS was 14.9 months (95% CI: 7.6–21.7 months; **Table 2**; **Figures 2**, **3**). In addition, the median DOR was 3.4 months (**Table 2**, **Figure 4**). In the sintilimab plus fruquintinib group, the ORR was 7.4% and DCR was 62.9%. The PFS and OS were observed at 3.8 months and 11.7 months, respectively. As shown in **Figure 5**, there was 1 patient with PR in liver metastases and another patient with SD in multiply lung metastases.

**Table 2:** Response Outcome.

Outcome	N = 45
ORR (CR+PR),%	11.1%
DCR (CR+PR+SD), %	62.2%
DOR, median	3.4m
PFS, median (95% CI)	3.8m (2.8 - 4.8)
OS, median (95% CI)	14.9m(7.6 - 21.7)

Based on the results of univariate analysis, it was found that PFS for left and right primary tumors was not statistically different (*p* = 0.946) and PFS with or without lung metastases was not statistically different (*p* = 0.835) as well. As shown in **Tables 3**, **4**, neither KRAS mutation nor liver metastases were associated with a longer PFS or OS. The same trends were observed from the results of multivariate analysis.

**Table 3:** Univariate analysis and multivariate analysis of clinical variable for the prediction of progression free survival.

Variable		Univariate analysis		Multivariate analysis	
		HR	p	HR	p
Gender	Male vs Female	0.794 (0.415 - 1.519)	0.486	0.837 (0.304 - 2.309)	0.731
Age median =54	<54 vs >=54	0.846 (0.456 - 1.570)	0.596	0.772 (0.322 - 1.850)	0.562
ECOG PS	0-1 vs >=2	1.214 (0.605 - 2.437)	0.586	1.023 (0.413 - 2.535)	0.960
Tumor location	Left vs Right	1.024 (0.517 - 2.027)	0.946	1.448 (0.607 - 3.454)	0.404
Histological differentiation	Poorly vs Moderately vs Well	1.938 (0.455 - 8.260)	0.371	1.370 (0.348 - 5.391)	0.653
Kras status	Wild vs Mutant	1.390 (0.746 - 2.589)	0.300	2.524 (0.918 - 6.942)	0.073
Number of metastatic organs	<=2 vs >2	0.891 (0.479 - 1.656)	0.715	0.835 (0.383 - 1.819)	0.649
Liver metastasis	Yes vs No	0.546 (0.250 - 1.191)	0.128	0.494 (0.194 - 1.255)	0.138
Lung metastasis	Yes vs No	0.936 (0.505 - 1.737)	0.835	1.427 (0.571 - 3.568)	0.446
Prior surgery	Yes vs No	1.324 (0.660 - 2.656)	0.429	1.866 (0.691 - 5.044)	0.219
Prior Bevacizumab	Yes vs No	0.827 (0.321 - 2.129)	0.694	1.851 (0.538 - 6.366)	0.329

**Table 4:** Univariate analysis and multivariate analysis of clinical variable for the prediction of overall survival.

Variable		Univariate analysis		Multivariate analysis	
		HR	p	HR	p
Gender	Male vs Female	0.561 (0.253 - 1.244)	0.155	0.525 (0.137 - 2.017)	0.348
Age median =54	<54 vs >=54	1.086 (0.521 - 2.267)	0.825	1.045 (0.419 - 2.606)	0.924
ECOG PS	0-1 vs >=2	0.643 (0.271 - 1.527)	0.317	0.271 (0.090 - 0.811)	0.020
Tumor location	Left vs Right	0.955 (0.432 - 2.112)	0.910	2.123 (0.688 - 6.549)	0.190
Histological differentiation	Poorly vs Moderately vs Well	0.891 (0.279 - 2.845)	0.846	0.330 (0.075 - 1.452)	0.143
Kras status	Wild vs Mutant	0.815 (0.351 - 1.893)	0.635	1.934 (0.546 - 6.844)	0.307
Number of metastatic organs	<=2 vs >2	0.710 (0.329 - 1.533)	0.384	0.384 (0.129 - 1.140)	0.085
Liver metastasis	Yes vs No	0.633 (0.240 - 1.671)	0.356	0.445 (0.118 - 1.673)	0.231
Lung metastasis	Yes vs No	1.297 (0.602 - 2.795)	0.507	1.435 (0.468 - 4.404)	0.528
Prior surgery	Yes vs No	2.030 (0.862 - 4.777)	0.105	3.699 (1.021 - 13.401)	0.046
Prior Bevacizumab	Yes vs No	0.770 (0.178 - 3.323)	0.726	0.983 (0.169 - 5.737)	0.985

Out of the 45 patients, 62.2% patients had grade 1-2 treatment-related adverse effect(TRAE) and 6.7% patients had grade 3 TRAE. No treatment-related death was reported. The most common grade 1-2 TEAEs included hypertension (22.2%), fecal occult blood positive or bleeding (17.8%), hand-foot syndrome (13.3%), hypothyroidism (8.9%), and hepatitis (4.4%). One patient suffered from pancreatitis.

## Discussion

Patients with mCRC will eventually face challenges in their medical management following the failure of standard treatment. Fortunately, there were several choices with good performance in third or further line therapy. Previous research has indicated that immunotherapy showed no effect on mCRC patients with MSS (19), possibly due to the complex tumor microenvironment that counteracts antitumor immunity *via* a combination of low antigenic tumor cells and an immunosuppressive tumor microenvironment (20). However, the REGONIVO study demonstrated a promising anticancer activity in mCRC patients with MSS who were administered with a PD-1 inhibitor (nivolumab) and regorafenib, which exerted a profound impact on the treatments for mCRC patients. In addition, three additional antibody therapeutics developed by Chinese companies (tislelizumab, sintilimab, and camrelizumab) became available in China in January 2020 (21), all of which have been proved to be highly selective, fully-humanized monoclonal antibodies that have a potential in blocking the interaction between PD-1 and its ligands (22). Based on the REGONIVO combination strategy, our center attempted a similar regime using fruquintinib combined with several PD-1 inhibitors as a third-line therapy for mCRC patients with MSS. Therefore, we retrospectively reviewed those patients and analyzed the efficacy of their treatments.

We obtained medical records from 45 patients for statistical analysis. Generally speaking, our therapy regime showed certain therapeutic efficacy for the patients. In this study, DCR was 62.2%, and ORR was 11.1%, with 4 PRs observed. Comparatively, in the REGONIVO study, ORR was observed in 40% of patients and 8 out of 50 patients (including 25 CRC and 25 gastric cancers) had PRs, resulting in an ORR of 33% in MSS CRC patients. In addition, our response rates were not better than those of the REGONIVO research. The efficacy of the combination treatment in the North American population differed from that in the Japanese group. More specifically, in the North American version (13) of REGONIVO, 5 patients (7.1%) out of 70 had a PR and 22 (31.4%) had SD. The LEAP-005 study (12) (NCT03797326) evaluated the efficacy and safety of lenvatinib combined with pembrolizumab in patients with previously treated CRC, and found an ORR of 22% (95% CI: 9–40) and a DCR of 47% in 32 CRC patients. The reason for the discrepancies between studies might lie in the fact that the REGONIVO study was a dose-finding and dose-expansion phase 1b trial, which was aimed to exploring the safety and recommended doses. Thus, the response rates should be further verified based on larger cohorts in future. Second, the efficacy of the combination treatment might also vary with the specific anti-PD-1 or angiogenesis agent and the population. Third, patients in trials usually have PS scores of 0-1. Considering real-world studies do not limit PS scores, the patients in our study had worse PS scores, which may have affected the treatment responses. Furthermore, the data from LEAP-005 and REGONIVO are certainly not definitive and that randomized phase 3 trials are ongoing with data pending to determine the true efficacy of these regimens.

Compared to the PFS of 6.3, 3.7 and 2.0 months in the REGONIVO (6), FRESCO (23), and TAS-102 study (5), the median PFS in our study was 3.8 months, which was not superior to that of the REGONIVO study but similar to other studies associated with third or subsequent line treatment for mCRC. We acknowledged the non-superior PFS of fruquintinib and anti-PD-1 treatment in this study since real-world studies were usually very complex involving a number of factors that reflect actual clinical practice, whereas clinical trials were able to exclude poor conditions. Furthermore, although regorafenib and fruquintinib were the same types of oral anti-angiogenesis agents, regorafenib was multi-targeted (7)and fruquintinib was highly selective for VEGFR1, VEGFR2, and VEGFR3 (17), which suggests that their underlying mechanisms at the functional site are different. In addition, the molecular properties of PD-1-targeted antibodies might be another influencing factor. We acknowledged the limitations in comparing nivolumab and other types of PD-1 antibodies. However, nivolumab and pembrolizumab differed in their binding sites’ extent and spatial location with the flexible PD-1 loops (24). Based on the available characterization data on anti-PD-1 antibodies, the molecular behavior between nivolumab and other anti-PD-1 antibodies might differ from each other (25, 26). We also considered, to some extent, the dose effect of fruquintinib on its efficacy. Since the recommended dose of fruquintinib on a continuous regimen was 5 mg per day, some patients in our study were administered 3 mg due to their tolerance (27, 28). Therefore, the dose may have affected clinical efficacy as well.

In addition, we compared our results with published data about fruquintinib combined with sintilimab in the treatment of mCRC. In our study, the ORR was 7.4% and DCR was 62.9%, and the PFS was observed to be 3.8 months in the sintilimab plus fruquintinib group. Comparatively, as reported by Jin Li et al. (16) in a phase Ib/II, multicenter, two-stage study, the ORR was 27.3% and 18.2% in the 5 mg-intermittent and 3 mg-continuous groups, respectively, and the PFS was 6.8 and 4.3 months for the 5 mg-intermittent and 3 mg-continuous groups, respectively, which seemed to be better than that in our study. Thus, it could be inferred that the benefit from the immunotherapy plus TKI agents was less concerning the complexity of the background in the real word study.

Notably, compared to 9.3 months for FRESCO study, which was the longest OS that has ever been reported, the median OS in our real-world study was much higher, reaching up to 14.9 months. Since we cannot directly compare the efficacy of our research with the FRESCO or REGONIVO studies, their difference in OS needs to be further validated in a larger group.

The PFS in our study appears to be no better than the results from the FRESCO study. Accordingly, we further assessed whether clinical characteristics were correlated with clinical outcomes. Gender, tumor location, metastatic organs, and KRAS status were not significantly associated with PFS, which was inconsistent with the result of REGONIVO study that all patients responding in the REGONIVO study (6) were male with lung metastases and had PS scores of 0. Therefore, the results of REGONIVO study may have been biased by the small sample and need to be further confirmed in a larger population.

One shortcoming of our study was the limited sample size and data on PD-L1 expression. PD-L1 was found to respond to anti-PD-1 antibodies in several tumors (29). However, there was little data on PD-L1 expression in our study. Thus, the role of PD-L1 as a potential biomarker for CRC patients could not be fully evaluated. Besides, the data on immune checkpoints (such as CTLA4), which potentially attenuate anti-tumoral immune responses and facilitate tumor growth and metastasis (30), were absent in our study since there were only a few cases treated with CTLA4 in our center. In addition, our study included various types of PD-1 antibodies, which was challenging to determine which PD-1 antibody was the best in combination with Fruquintinib in the treatment of mCRC.

In conclusion, we found that fruquintinib, in combination with anti-PD-1, had clinical activity in mCRC refractory to standard chemotherapy. Nevertheless, this benefit was not observed across all prespecified subgroups of patients. Further research would be necessary to assess OS benefits based on a larger sample size in future.

## Data Availability Statement

The raw data supporting the conclusions of this article will be made available by the authors, without undue reservation.

## Ethics Statement

The studies involving human participants were reviewed and approved by the independent ethics committee of Chinese PLA General Hospital. The patients/participants provided their written informed consent to participate in this study.

## Author Contributions

MG, NQ, YZ are in charge of data collection and writing. HY and HS contribute to data collection. ZW and GD come up thoughts. All authors contributed to the article and approved the submitted version.

## Conflict of Interest

The authors declare that the research was conducted in the absence of any commercial or financial relationships that could be construed as a potential conflict of interest.

## Publisher’s Note

All claims expressed in this article are solely those of the authors and do not necessarily represent those of their affiliated organizations, or those of the publisher, the editors and the reviewers. Any product that may be evaluated in this article, or claim that may be made by its manufacturer, is not guaranteed or endorsed by the publisher.
